# Remote Supervision in Short-Term Global Health Experiences

**DOI:** 10.1155/2018/5629109

**Published:** 2018-10-02

**Authors:** Pryanka Relan, Kristy C. Y. Yiu, Henry C. Lin, Lawrence C. Loh

**Affiliations:** ^1^Johns Hopkins Bloomberg School of Public Health, 615 N Wolfe Street, Baltimore, MD 21202, USA; ^2^Faculty of Health Sciences, McMaster University, 1280 Main Street West, Hamilton, ON, Canada; ^3^The 53rd Week Ltd, 116 Kent Avenue Suite 116, Brooklyn, NY 11249, USA; ^4^Dalla Lana School of Public Health, University of Toronto, 155 College Street, Toronto, ON M5T 3M7, Canada

## Abstract

The global health development community is increasingly examining the phenomenon of short-term experiences in global health (STEGH), with an aim to mitigate the negative impacts of such activities on host communities. Appropriate supervision is one strategy, but various barriers (e.g., institutional requirements) limit the availability of qualified supervisors. Remote supervision represents one potential model to provide supervision that may mitigate the negative impacts of STEGH. This paper reports observed outcomes from a description of a pilot remote supervision program employed in a global health program for Canadian undergraduate students. Benefits for learners included greater confidence and independence, greater perceived effectiveness in conducting their project abroad, and reassurance of remote support from their supervisor, supplemented with day-to-day guidance from the local partner. Host communities reported greater trust in the bidirectional nature of partnership with the visiting institution, empowerment through directing students' work, and improved alignment of projects with community needs. Finally, faculty noted that remote supervision provided greater flexibility and freedom when compared to traditional in-person supervision, allowing them to maintain professional duties at home. Collectively, this pilot suggests that remote supervision demonstrates a potential solution to mitigating the harms of STEGHs undertaken by learners by providing adequate and appropriate remote supervision.

## 1. Introduction

Short-term experiences in global health (STEGH) continue to increase in frequency and popularity, particularly among premedical undergraduate students, medical trainees, and young professionals [1]. One notable contributor to this trend has been increasing abroad-based educational programs provided by universities and medical schools, variously defined as “alternative spring breaks,” “study abroad semesters,” “medical missions,” “volunteer trips,” and the like.

The increasing popularity of STEGH has also identified concerns around the negative impacts of such efforts on host communities and a need to identify best practices to support the responsible conduct of such educational programs. Specific concerns described in global health and development literature include cultural incongruence, perpetuation of power differentials, and the cultivation of dependence on foreign-service learners which disrupt local systems and offset short-term gains provided to these communities [2–4].

Literature has highlighted various strategies to address these concerns, including the incorporation of cross-cultural effectiveness and cultural humility training into educational programs [1] and appropriately preparing and engaging learners ahead of their participation [5–7]. Another strategy commonly mentioned is appropriate supervision of participants. However, in many cases, STEGH occur without adequate or appropriate on-the-ground supervision for learners [8]. This often could be attributed to an inadequate number of available supervisors. In academic institutions, this deficit of appropriate supervisors is typically driven by a lack of faculty members with interest or experience, difficulty in securing time away for said faculty, institutional policies, or other constraints, such as financial or personal barriers to faculty participation.

One potential strategy to remotely supervise students could be through e-mail communication. However, supervision through e-mail communication is asynchronous and prevents the learner from receiving immediate feedback, which can be detrimental in some situations. Literature has also found that e-mail-based supervision is “unclear or not conducive to thoughtful transfers” [9, 10] and open to misinterpretation [11]. This limits the use of e-mail as a viable option for supervision.

In recent years, improved Internet and technology have permitted remote supervision as a potential solution to these barriers. Combining regular check-ins via Internet telephony (e.g., Skype) and more regular supervisory direction from a local partner, we propose that an effectively deployed remote supervision model might enable qualified faculty to remotely supervise learners abroad.

While extensive research has been conducted on the traditional models of successful faculty supervision and mentorship in other disciplines, there is a lack of literature addressing the potential of remote supervision for global health learners at the undergraduate and graduate levels [12–15]. As part of conducting a quality review, this process paper describes the components implemented and informal feedback received around a novel remote supervision pilot for undergraduate learners abroad. We identify successes and challenges encountered with the model and propose opportunities for future evaluation.

## 2. The Remote Supervision Program Pilot

The pilot remote supervision process being described involved a formal partnership between an academic institution and a nonprofit organization to provide specialized STEGH programming. Following student selection and enrollment, the nonprofit organization was responsible for providing predeparture training and remote supervision of the students during the course of the experience, in concert with a local on-site partner.

Students for the pilot program were enrolled in an undergraduate health sciences global health specialization program that incorporated “study abroad” experiences that occurred at various domestic and international sites over three to four months. During this experience, which typically occurred during the student's third year, the students live, volunteer, and integrate into a community site abroad, with the objective of gaining firsthand experience that underscores the complexity in global health and the importance of community health. Students are selected into specific experiences based on standardized criteria including academic achievement, background, and potential to be trained and deployed as effective resources in the field.

For the purposes of the remote supervision pilot, conducted over the three academic years between 2013 and 2015, students applied to be placed with a nonprofit organization in a community in Latin America. During each academic cycle, four global health students were selected by the university program. Students were asked to provide a motivational statement and curriculum vitae and respond to a written application with questions, all of which were assessed for interest in global health. Many of the graduates indicated a desire to pursue healthcare-related professional or graduate programs.

Selected students subsequently underwent predeparture training with an aim to involve them in locally directed projects with a community health focus on activities such as research or evaluation. They then travelled to the host community where they were jointly supervised by the nonprofit and a host community hospital. Three volunteer faculty members, affiliated with the nonprofit, provided academic supervision for the students from North America via remote supervision, with the leaders of a local hospital partner providing direct, on-the-ground student supervision. These faculty members were selected based on their relationship with the local partner in that country as well as past experience supervising undergraduate students on self-directed academic projects.

The program phases were broadly structured as follows:

- 8-week predeparture curriculum at home: nonprofit faculty met with the students in person for a two-hour long session to provide an overview to the program and initiate an 8-week, online predeparture curriculum. In addition to providing guidance around expectations and supervision via remote supervision, students undertook curriculum modules, as listed in Table 1, similar to other STEGH with traditional supervision models. These provided students with training in key concepts such as cultural humility and highlighted ethical considerations in volunteering abroad, while also conducting skills development (e.g., principles of program evaluation, assessment tools, or language training) and providing additional project details.  Table 2 outlines the full list of content areas taught. After each module, students were required to complete a written blog entry, which formed the basis of their evaluation. These were remotely reviewed by faculty, who provided directed feedback and remedial work as needed. For selected students, there was additional scrutiny given to their progress to ensure that they would be able to function independently abroad while being remotely supervised.
- 12-week on-the-ground program in host community: following their predeparture training, students arrived in the host community to begin their project. Faculty supervisors from the nonprofit were present during the students' initial week to facilitate their introduction to the local community and local partner leaders. At this time, the students also reconfirmed the broad concept of their project and initial steps. After the first week, nonprofit faculty returned to North America. Day-to-day direction and work were guided by local host partners, while nonprofit faculty supervised the academic aspects of the student's experiences through weekly, two-hour long Skype meetings to discuss project work and facilitate updates, debriefs, and discussions of ethical and logistical challenges arising on the ground. Specific details about project course, timeline, data points, and logistics were frequently discussed. All check-in sessions were conducted jointly with the local supervisors to provide a relevant context.
- 4 weeks after return home: a final debrief between students and faculty was organized to finalize the project deliverables, identify next steps for the project, and address any problems that may have arisen at the end of the project or during the transition home.

Student projects were identified and developed by the local partner on the basis of community health and development priorities. For example, one group of students conducted a needs assessment to inform sexual health educational programs among adolescents in the target community. The daily work of students undertaken to support this project included survey distribution and collection, interviews with key stakeholders, review of educational curricula, and thematic analysis. The accumulation of these tasks resulted in an overall report that would support sexual health educational efforts undertaken by the local partner agency.

## 3. Observations

Over the course of this three-year pilot, eleven students participated in the remote supervision pilot program. These observations are based on a review of notes from check-in distance meetings as well as informal debrief discussions that were held upon returning home. Findings from this review have justified the implementation of the pilot as an ongoing program with a formal evaluation mechanism and are summarized below.

The general view from the pilot years were positive. In particular, it was suggested that collaboration with local partner leaders allowed for a combination of strategic academic leadership and supervision provided by remote faculty with the local partner leaders providing direct daily oversight of operations and completing tasks. In debrief meetings, students expressed that weekly check-ins via Skype provided an opportunity to track and monitor projects and issues around logistics (e.g., security and safety). In one pilot year, it was seen that an early check-in session identified a key area of focus for the students' data collection strategies. This then formed the foundation to identify strategies to address gaps in collected data and next steps that tracked through discussions in subsequent check-in sessions.

The review of pilot findings identified several potential benefits that may be borne out upon formal program implementation. Involved students mentioned that the absence of full-time faculty on the ground meant that they had the opportunity to gain confidence and independence in interacting with the local partner and community. However, they did not do so alone, but with the reassurance that remote faculty would be available both for regular check-ins and for ad hoc requests. An added benefit was that local leadership was empowered to be more involved in directing the work of students on day-to-day activities and supporting students in navigating their way through the local cultural context.

Remarks also suggested that having a remote supervisor familiar with the program, community, and work also allowed students to benefit from their insight into how their work fit into the overall partnership with the community while facilitating their understanding of the cultural implications of their participation. Students across all three pilot years expressed their belief that this led to greater effectiveness in their work, and in debrief notes our findings matched those of another qualitative study that found remotely supervised students may be more receptive to reflection than traditionally supervised students, in that they are able to communicate from the comfort and privacy of their selected location [16].

Insights from faculty notes and reports on students suggested their belief that learners in the pilot seemed more willing to engage in the concepts around their project work, and this was particularly exemplified in their conversations during the post-return debrief. Faculty also expressed some incidental benefits from the pilot, including greater flexibility and freedom to remotely supervise learners abroad while maintaining their professional and academic duties back at their home institution. These insights also suggested that dividing and assigning academic and on-site supervisory roles with community leaders permitted supervision of learners in a more holistic manner.

From the notes on discussions with community leaders through the pilot, host partners observed that employing a remote supervision model that necessitates their taking on a role as day-to-day primary supervisor fosters greater trust and commitment to a bidirectional partnership with the visiting academic institution when compared with more traditional models. This suggested that a division in supervision would empower community leaders to involve students in established priority projects and match their project with true local needs. Local leaders also suggested that the remote supervision model helped to foster a greater sense of leadership and autonomy over the work of the student learners on their various community projects.

## 4. Discussion

Medical schools and universities are increasingly looking for strategies to provide adequate supervision to learners on study abroad programs to ensure such programs proceed in a responsible manner and provide new opportunities for a growing number of learners. Our pilot study has demonstrated that remote supervision could play an integral role in the conduct of STEGH and in fulfilling this need. This is particularly salient as employing traditional supervision models on a larger scale is proving to be increasingly challenging in the face of growing interest in STEGH; bringing groups of students abroad on repeated, frequent visits is not the primary role for most academic faculty.

Remote supervision is a possible solution to the increasing demand to accommodate for more students in STEGH without placing additional burden on faculty and institutions. If appropriately deployed, remote supervision could obviate the need for faculty to commit to repeated trips with learners, but in fact could allow faculty to cosupervise multiple learners at multiple sites remotely while fulfilling their primary academic responsibilities at home. Learners in this model are enabled by technology to ask questions of remote faculty in real time, with concurrent guidance from the local partner, to more responsibly participate in their STEGH as compared to an unsupervised experience.

Remote supervision is an efficient and flexible option for supervising STEGH learners, provided an effective model is selected. In reflecting on what constitutes an effective model, our pilot also identified key themes suggesting that a successful remote supervision program for STEGH requires a combination of* appropriate* and* adequate* supervision of a student that has received adequate* preparation*.

As demonstrated by our program, the provision of* appropriate* supervision means that supervising remote faculty should ideally have experience in the locale that students are deployed to, or similar locales if a complete match is not possible, and a relationship with the local partner involved in supervision. In our pilot program, existing faculty members had worked in sites where students were sent and were familiar with the programs as well as the contact personnel on the ground abroad. This facilitated remote supervision for students and helped to mitigate challenges and optimize the outcomes of project work for the host community and the learner.

*Appropriate* supervision, beyond ensuring students' academic and safety needs are met, includes accounting for student needs and preferences for frequency and intensity of supervision. Studies show that students prefer face-to-face interactions when dealing with more detailed queries and assistance. This was previously difficult to achieve with traditional distance supervision (e.g., via e-mail) [10]. Remote supervision, however, provided an avenue for participants to do so, despite the physical distance, by taking advantage of video conferencing technologies like Skype or FaceTime.

We also contend that appropriate supervision includes at least some face-to-face meetings during the course of the preparation, even if the bulk of the experience is supervised via remote supervision. In this, the initial in-person meeting and first week visit by faculty helped students and faculty develop a supportive rapport. This observation is supported by previous literature on remote supervision within a nursing context where face-to-face meetings were found to be crucial in establishing an open and trusting relationship [17].

Ensuring* adequate* supervision is also critical, given that the remote supervisor is not physically present with the learner throughout their experience. To help guide situations and unfamiliar territory with sometimes necessary immediacy, our pilot identified the need of having a local supervisor available for questions during local hours, who would be quickly available to learners in order to resolve urgent issues arising on the ground, provide cultural brokering services, and defray any cultural challenges as they arise. In this manner, the initial visit of the remote supervisor at the host site also helped to delineate roles between the remote and local supervisor for learners. Done early on, this helped to facilitate a smooth transition to the joint remote supervision model for the remainder of the learners' experience.

*Preparation *is an element of successful STEGH as a whole and arguably even more critical for STEGH facilitated by remote supervision. Predeparture preparation should include guidance specific to preparing students on this novel supervision, techniques, and the benefits and challenges that it provides. As demonstrated in our pilot program, this includes training on roles and responsibilities in the cosupervision model and preparation for potential backup communications in the event of a disaster or Internet outage. Though remote supervision in our program was Internet-based, it was critical to ensure that mobile technology was also available as a backup method for learners to reach out to remote supervisors.

Considering these three elements, a* prepared* student undertaking a STEGH with an* adequate, appropriate* remote supervision program in place not only offers significant benefits to the student, remote faculty, and local partners involved, but adds a unique dimension to the depth of the abroad experience overall. In the absence of a concurrently visiting faculty member, students are able to gain a new level of independence in their work and have a unique opportunity to develop cross-cultural competence. Distance present in a supervisory relationship may help diminish the perception of power differential between the student and faculty, making it easier for the two parties to connect and develop an open and collegial relationship [18].

These three key elements of success identified by our pilot arose from a combination of factors. This included having a reliable local partner (fostered by a four-year-old partnership between the academic institution, the nonprofit organization, and the host community hospital), carefully selecting students who demonstrated great potential for independent study based on prior academic achievement and standardized criteria (e.g., motivation, second language skills, etc.), a clearly delineated project for which students prepared extensively for, prior to departure, a robust predeparture training program (Tables 1 and 2), an initial on-site visit by the faculty supervisor followed by regularly scheduled check-in sessions, and stable and consistent access to the relevant Internet technology to facilitate communications.

One area for further development included the ideal frequency for communication with the remote faculty. In particular, our pilot favored twice or three times a week communication, though daily connections may have provided remote faculty with more regular updates around the tasks or location of all students at all times. In settings where the local leadership may not be as experienced, this could pose challenges, and more frequent remote check-in sessions may be required.

On balance, remote supervision seems to be a promising alternative to in-person supervision. It is worth noting that other fields outside of global health education have published numerous benefits noted from their own explorations of remote supervision. One study by Bertsch et al. noted that remote teaching via video conference had similar successes to in-person classroom instruction for medical students in clerkship [19]. Among medical specialties, remote supervision is increasingly used in teaching procedural-based specialties such as surgery and dermatology [20, 21].

## 5. Future Directions

Although we focused on remote supervision for a 12-week undergraduate study abroad STEGH experience, this concept may also be expanded to other disciplines, such as medical students and other graduate students on electives or field work that might be conducive to remote oversight. Other ideas to be explored in future iterations of this project include possibly a peer-learning model, where students abroad on STEGH can share their experiences with other students at their home institution in real time.

Remote supervision with different organizations and institutions is another future possibility, as is the deployment of remote supervision in even more remote settings. Finally, one can also consider the model of “participatory remote supervision” as practiced at one North American institution. This model involved faculty work alongside the learners as collaborative knowledge builders, where “they ‘build-on' students' notes, enter notes of their own in views, construct new views, reference different notes, or create synthesizing ‘rise-above' notes” [22]. Participatory remote supervision could allow learners to benefit from the contributions of multiple remote faculty supervisors and also provides remote faculty with a better picture of the work as it proceeds. It also transitions faculty from being “experts” transmitting knowledge towards more collaborative roles that minimizes the hierarchical distance between the faculty and learner [23].

This paper has highlighted the various observations around the deployment of a pilot remote supervision program for North American learners engaged in STEGH and reviewed exciting potential directions. Although the findings presented are preliminary, they justify a more formal implementation and evaluation of the program to fully capture the outcomes of this model and determine the optimal format in which such experiences should occur. Success in this regard will provide additional detail into how remote supervision can be made a viable option for institutions, host communities, and learners engaged in STEGH.

## Figures and Tables

**Table 1:** Predeparture manual outline.

**Section **	**Title**
**1**	Introduction to the organization – mission statement, core values, history

**2**	Projects – Medical Service Initiatives, Collaborative Web-Based Platform, Girl Rising: Adolescent Hygiene and Menstruation Education, Embedded Learning Experience, Active Research Projects

**3**	Target Community and Partners – basics about the Domincan Republic, La Romana, The Bateyes, and Good Samaritan Hospital

**4**	Medical Service Initiatives – Overview, Goals, How the Clinics Work (including Triage, Clinic and Pharmacy information), Setting up the Clinic, Daily Clinic Routine

**5**	Introduction to the 53rd week team – Individual bios of all directors and managers

**6**	Pre-trip preparation list – Airline tickets, passports, government registration and consulate information, visas, travel insurance, immunizations, prophylaxis, accomodations and meals, cell phones, packing list

**7**	Contact Information

**Table 2:** Curriculum summary.

**Section**	**Title**
**1**	Background about the locale - - Economic, social, cultural indicators and considerations; Rationale for work in this geographic area

**2**	Needs Assessment and Environmental Survey/Scan - - Key components; How to build and implement, including logistical considerations of carrying out the surveys in each setting

**3**	Content/Project-Specific Considerations

**4**	Project Planning and Sustainability

**5**	Basic Teaching Skills

**6**	Cultural Competency

**7**	Global Health Ethics

## Data Availability

The data used to support the findings of this study are included within the article.
